# The three-stage assessment to support hospital–home care coordination in Tshwane, South Africa

**DOI:** 10.4102/phcfm.v12i1.2385

**Published:** 2020-07-07

**Authors:** Jannie F.M. Hugo, Tshegofatso C.R. Maimela, Michelle N.S. Janse van Rensburg, Jan Heese, Chitalu E. Nakazwa, Tessa S. Marcus

**Affiliations:** 1 Department of Family Medicine, Faculty of Health Sciences, University of Pretoria, Tshwane, South Africa; 2 COPC Research Unit, Faculty of Health Sciences, University of Pretoria, Tshwane, South Africa; 3 Department of Public Health Medicine, Faculty of Health Sciences, Steve Biko Academic Hospital, Tshwane, South Africa

**Keywords:** Care coordination, Three-stage assessment, Collaborative care, Down referral, Patient discharge, Interprofessional and intersectoral networks

## Abstract

**Background:**

In complex health settings, care coordination is required to link patients to appropriate and effective care. Although articulated as system and professional values, coordination and cooperation are often absent within and across levels of service, between facilities and across sectors, with negative consequences for clinical outcomes as well as service load.

**Aim:**

This article presents the results of an applied research initiative to facilitate the coordination of patient care.

**Setting:**

The study took place at three hospitals in the sub-district 3 public health complex (Tshwane district).

**Method:**

Using a novel capability approach to learning, interdisciplinary, clinician-led teams made weekly coordination-of-care ward rounds to develop patient-centred plans and facilitate care pathways for patients identified as being stuck in the system. Notes taken during three-stage assessments were analysed thematically to gain insight into down referral and discharge.

**Results:**

The coordination-of-care team assessed 94 patients over a period of six months. Clinical assessments yielded essential details about patients’ varied and multimorbid conditions, while personal and contextual assessments highlighted issues that put patients’ care needs and possibilities into perspective. The team used the combined assessments to make patient-tailored action plans and apply them by facilitating cooperation through interprofessional and intersectoral networks.

**Conclusion:**

Effective patient care-coordination involves a set of referral practices and processes that are intentionally organised by clinically led, interprofessional teams. Empowered by richly informed plans, the teams foster cooperation among people, organisations and institutions in networks that extend from and to patients. In so doing, they embed care coordination into the discharge process and make referral to a link-to-care service.

## Introduction

South Africa’s current public healthcare delivery model seeks to achieve improved health outcomes by employing safe, accessible and efficient quality services.^1,2,3^ The system is organised hierarchically with primary healthcare (level 1) rendered in districts by district hospitals, community health centres (CHCs) and clinic facilities as well as through community outreach teams and ambulance services. Specialist and subspecialist care (levels 2, 3, 4) is delivered provincially by regional, tertiary and central hospitals.^4,5,6^ Schematically, this tiered hierarchy is intended to structure referral pathways. Referral thus is an intentional systems design feature to enable service continuity and ensure access to and cost-effective use of public healthcare services.^7^ In institutionally complex health settings, referral pathways based on reasonable clinical processes seldom achieve health system responsiveness, organisational efficiency and improved population health outcomes^8,9,10^ without proactive and deliberate care-coordination.

Care-coordination is the facilitation of ‘the right healthcare services in the right order, at the right time and in the right setting’.^11^ Practically, the purposive linking of patients to appropriate places, services and people is simultaneously sequential and parallel.^11^ In sequential care-coordination, services and information flow upwards from acute or routine and general through complicated to complex specialist curative and rehabilitative clinical care. The downward sequence is less ordered or predictable as patients may flow out – as in ‘discharge home’ – and/or down to regional or district hospitals, primary health care (PHC) clinics and services that extend to the home.Parallel coordination centres on people, roles and plans. It is also structurally shaped by the way services are organised through hierarchy or compartmentalisation between departments and firms within facilities or between sections and services within PHC.

Referral critically depends on relationships between providers, patients and caregivers, as well as care adapted to patients’ needs and contexts. It is enacted through interpersonal, management, informational and longitudinal continuity.^11^ Continuity, thus, is embedded in the practice of sequential and parallel care coordination. Paradoxically, although the imperative of relationships for effective integrated healthcare^12^ ‘goes without saying’, and cooperation and collaboration are deemed to be important system values,^13,14,15^ they are not organised as intentional practices. As a consequence, functional, interprofessional care-coordination is often absent and contributes to poor clinical outcomes.^8,12^ The lack of parallel coordination simultaneously subverts essential relationships with individuals and families in their homes and communities,^16,17,18^ as well as sequential care pathways within and between levels and services.^16^

Notwithstanding the role and contribution of family physicians to care-coordination within primary care teams and between referral hospitals,^16,19^ comprehensive patient-centred, team-based coordinated care across all levels has been minimally explored in South Africa. The few models that have been described^9^ do not account for organisational and contextual factors that influence care-coordination, interprofessional cooperation and intersectoral- and service collaboration.^11,20^ Thus, in Tshwane, patient referral is seen as contributing to an ever-increasing service burden without commensurate resourcing. It is thought to contribute to bottlenecks and inefficiencies and impacts negatively on patient outcomes^21^ as well as the quality of care provided at all levels and in all facilities and services.^22^ Even though they are critical to the effective management of South African health in the context of the quadruple burden of disease, the interfaces between community-based and facility services are inconsistent and weakly supported^24^ and, as elsewhere, downward referrals from facilities to the community are the exception rather than the rule.^24,25,26,27^

Given the very limited understanding of the organisational and practice changes in care-coordination needed to improve health outcomes,^16,17^ in 2019, the Departments of Family Medicine and Clinical Public Health (University of Pretoria) initiated an action research intervention to facilitate interprofessional service delivery across clinical and community settings in the public sector in Tshwane, Gauteng. This article presents the results of the initiative’s initial downward coordination of care in the sub-district 3 (Tshwane District) public health complex.

## Setting

Coordination of care interventions are undertaken at Steve Biko Academic Hospital (SBAH), Tshwane District Hospital (TDH) and Tshwane Rehabilitation Hospital (TRH) in Tshwane District (Gauteng).

Steve Biko Academic Hospital is a central hospital. It is the referral centre for four district hospitals (TDH, TRH, Pretoria West Hospital [PWH] and Mamelodi Hospital), as well as surrounding PHC clinics, CHCs and related services. As a central hospital, SBAH also provides highly specialised services to patients from around the country, with a considerable number coming from Mpumalanga and Limpopo provinces. Demand for services at SBAH depends on the functioning of the health facility network it serves.

Tshwane District Hospital is a district hospital located 800 metres (m) from SBAH. It is the referral hospital for surrounding PHC clinics, CHCs and other PHC services.

Tshwane Rehabilitation Hospital is a rehabilitation hospital offering in- and out-patient services to adults and children in Gauteng and neighbouring provinces.^28^ It provides comprehensive physical rehabilitation services for patients with stroke, spinal cord injuries, amputation, head injuries and other neurological conditions.

All three facilities are teaching hospitals affiliated to the University of Pretoria (UP).^29^ As such, students and staff are exposed to community oriented primary care (COPC). It is an internationally accepted approach to healthcare where comprehensive, equitous, person-centred services are rendered along the home-facility-service continuum to individuals and families living in defined geographical areas. Through COPC, hospitals, clinics and ward-based outreach teams (WBOTs) are structurally linked to support information and service continuity. Comprehensive care integrated around health-service users is built through relationship continuity to enable effective case management.^30^

**FIGURE 1:**
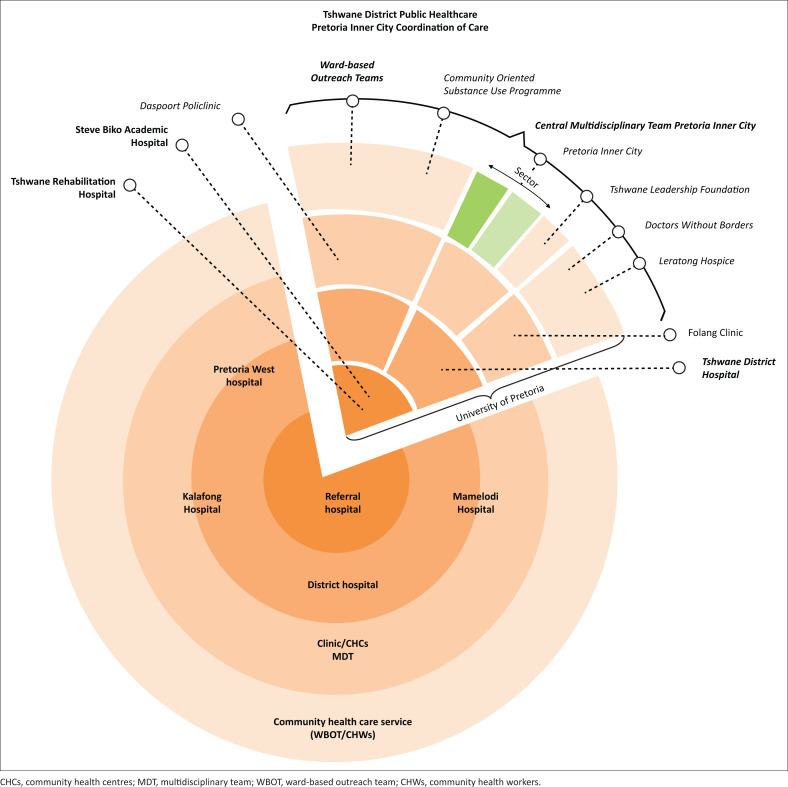
Service continuity in the Pretoria Inner City slice requires multi-layered, multi-level interactions from level 1 to level 4.

Institutionally, patient care is determined by the therapies required and the facilities and services available. Care-coordination is about ensuring that service pathways are both effective and efficient. This means working with patients, families and healthcare practitioners to provide the best services at the right level at the right time. In the public sector pie (Figure 1), service continuity in the Pretoria Inner City slice requires multi-layered, multi-level interactions from level 1 to level 4. Thus, coordination of care involves government, for example, WBOTs, City of Tshwane Community Oriented Substance use Programme (COSUP); not for profit organisations (NPOs), for example, Medicines Sans Frontier, Tshwane Leadership Forum; community-based organisations (CBOs), for example, Folong clinic, the PHC facility layer in the service hierarchy (Level 2), TDH and TRH (Level 3) and SBAH (Level 4). Designated individuals are responsible for care-coordination at each level, while the composition of multi-disciplinary teams changes according to need and availability.

## Research methodology and method

The research methodology follows a novel capability approach to learning. It assumes systems to be complex adaptive; processes to be guided by effective learning principles; and people to be agentic. The research sequence involves identifying the problem, issue or task, reviewing options and acting upon them (implementing the action plan). Within it, reviewing options is a four-dimensional iterative process that involves clarifying (reviewing), making sense of (reflecting), finding out about (reading, getting information) and working out possible ways to respond (re/act) to the issue or task.^31^ The capability approach incorporates many of the tenets of participatory action research (PAR) and quality improvement (QIP). It draws on the key PAR assumptions of strengthening relationships, creating involvement, developing capacity and transforming individuals and teams through experiential engagement, empowerment and the reciprocal transfer of expertise. It also draws on PAR methods, focusing on problem identification and the generation of solutions. However, it does not follow the PAR sequence of plan, act, observe, reflect. The capability approach also differs with the industrial assumptions that underpin the QIP ‘model for improvement’ and its sequence of plan, do, study, act.^32^

Using the construct of the ward round, interprofessional, multi-sectoral teams conducted ward rounds with a specific focus on care-coordination in SBAH internal medicine, the general and infectious disease wards at TDH, and at TRH. Participants in ward rounds included patients, clinicians, health and care professionals, researchers and service providers from the respective facilities, the University of Pretoria and relevant public, not-for-profit and community service partners.

Team composition and size during ward rounds varied by place, availability of participants and patient need. A core group, comprising academic clinicians as well as at least one representative of the ward clinical team involved in patient care at the respective facilities was present during all rounds. They were joined intermittently by professionals from relevant services as well as available clinical staff. An interdisciplinary team WhatsApp group was created to communicate patient-specific queries. In addition to providing information, it served as a platform for all team members to help make sense of and contribute to solutions, irrespective of ward round attendance.

## Sampling and data collection

Data were collected during 72 ward rounds conducted over the six month period April–September 2019. Ward rounds were conducted weekly on different weekdays at the three hospitals. Facility clinicians responsible for patient care purposively identified patients who they identified as being in need of care-coordination management support as their stay in the wards had exceeded hospital level norms (SBAH 6-8 days; TDH 4-6 days) and/or who they had been unable to refer or discharge (all three hospitals).

Data were generated through a clinician-led three-stage assessment and collaborative management planning with each patient.^32^ The three-stage assessment is understood as the most recent, justifiable comprehensive summary of the patient’s clinical, personal and social environment. Collaborative management planning focuses on the patient’s context and support; the immediate and near-term clinical actions; the possible steps that could be taken and the information, relationships and organisational support needed to realise patient centred clinical and care outcomes. To support information continuity in on-going care, patients were given the Road to Linked Care (RTLC), a patient retained record developed by UP Family Medicine, and a form based on the International Classification of Functioning, Disability and Health (ICF), developed by the World Health Organization (Collaborating Centre in South Africa for the Family of International Classifications [WHO-FIC]).^33^

Patient-specific data were collected by designated clinicians assigned to the ward-round team (two family medicine registrars and a public health medicine specialist). They used a data-collection rubric that covered date and time, hospital, patient unique identifier (i.e. hospital number, initials, ward and bed number), assessments (clinical, individual, and contextual), team involved, follow-up, hindrances and outcomes. Their hand written or electronic notes were then fed into an Excel sheet after the ward round. Ward round notes were supplemented by information retrieved from patient files.

## Analysis

The data-collection process yielded results that allowed for both quantitative and qualitative analysis. Quantitative data were captured on Excel and analysed using descriptive statistics. Qualitative data analysis was guided by the three-stage assessment and planning process generated in the ward rounds. Through a careful iterative reading of the assessment data, issues were grouped thematically^34^ according to similarities in their clinical management requirements.

### Ethical requirements

The study was approved by the UP Faculty of Health Sciences Research Ethics Committee (COPC 102/2011 as Amended 2016). Confidentiality and justice were assured by limiting care-related discussions to within the clinical team involved in patient management and by ensuring that patient plans were followed up. Patient data were anonymised for analysis purposes.

## Results

Results are presented under the following headings: (1) demographics, (2) the three-stage assessment and (3) parallel care-coordination.

### Demographics

A total of 94 patients were seen at the three hospitals over a period of six months. As seen in Table 1, 21% were from TRH, with the remainder being more or less evenly divided between SBAH (41%) and TDH (37%). Most (61%) were male, although comparing facilities, there were significantly more female than male patients at SBAH (*n* = 25, 64% female), while males predominated at TDH (*n* = 30, 86% male) and TRH (*n* = 14, 70% male).

**TABLE 1:** Number, age, sex and address of patients by facility.

		SBAH	TRH	TDH	Total
Patient	(*N*)	39	20	35	94
Age	(Mean)	50.6	40.6	47.1	-
Sex	M	14	14	30	58
	F	25	6	5	36
Address	Tshwane	23	11	16	50
	Gauteng	2	2	0	4
	Other province	4	6	4	14
	Other country	1	0	0	1
	Homeless	2	1	12	15
Discrepancy†		3	3	7	13

SBAH, Steve Biko Academic Hospital; TRH, Tshwane Rehabilitation Hospital; TDH, Tshwane District Hospital.

† , The difference between file record address and patient-reported place of residence.

The age of patients ranged from 5 to 95 years and averaged 46.9 (median = 47, SD = 19.0). Comparing facilities, patients at SBAH (mean age = 50.6, median = 55, SD = 18.9) and TDH (mean age = 47.1, median = 42.5, SD = 19.6) were slightly older than those at TRH (mean age = 40.6, median = 44, SD = 17.6).

In terms of addresses, hospital files yielded addresses for 84 (89.4%) patients, with data missing at both SBAH (*n* = 7) and TDH (*n* =3). In terms of geographical distribution, most patients (64%) gave Gauteng addresses, a little over a 10th (10.7%) were from other provinces, particularly Limpopo and Mpumalanga, and only one file had an address from outside South Africa. Analysed by facility, as expected, patients at TDH mostly had Tshwane addresses, while those at SBAH and TRH gave addresses from four of Gauteng’s five districts (Tshwane, Ekurhuleni, the West Rand and Sedibeng), as well as other provinces and other countries. At TDH hospital, homelessness was recorded as an address in eight instances. Based on the ward rounds across the facilities, 15 patients were found to be homeless. In 13 cases (15.47%), a discrepancy was noted between the address recorded on the file and that given by the patient during the ward round. Notably, none of these were from TRH. Also, in the course of the three-stage assessment, several patients said they lived in but were born outside South Africa.

### The three-stage assessment

In a three-stage assessment consultation, information is divided into three distinct sections – clinical, personal and contextual (i.e. environmental).

#### Clinical assessment

During the ward rounds, 178 clinical assessments were made for 94 patients (see Table 2). Thirty-five patients had a single item, 38 had two, 17 patients had three and four patients had four clinical-assessment items. The clinical assessments were grouped into 20 themes or areas according to similarities in their epidemiological and clinical management requirements. These themes were categorised by clinical diagnosis. Neurological or neurosurgical problems (31) were the most common, followed by infections other than human immunodeficiency virus (HIV) or tuberculosis (TB) (18), HIV-infection (17), cardiovascular (16), tuberculosis (16), personal (e.g. pressure ulcers) and social problems (11) and endocrine (notably diabetes), nephrology and substance use disorder (10 each, respectively).

**TABLE 2:** Clinical assessments – Item numbers by category per facility.

Clinical category (Clinical assessments as recorded during the ward round)	Total	SBAH	TDH	TRH
**Neurological or neurosurgical:** Acute disseminated encephalomyelitis (ADEM), cerebral vascular accident (CVA), delirium, Guillain-Barré syndrome (GBS), intracranial haemorrhage, paraplegia, post-neurosurgery, quadriplegia, subdural haemorrhage, syncope, traumatic brain injury (TBI)	31	7	6	18
**Infections (other than HIV/TB):** Abscess, chronic diarrhoea, chronic otitis media, cryptococcal meningitis, lower respiratory tract infection (LRTI), osteomyelitis, pneumocystis pneumonia (PCP), septic arthritis, septic eye prosthesis, septic shock, urosepsis, urinary tract infection (UTI), neurosyphilis	18	6	11	1
**Human immunodeficiency virus (HIV):** HIV	17	2	14	1
**Cardiology:** Atrial fibrillation (AF), cardiac failure, cardiovascular disease (CVD), dilated cardiomyopathy, heart failure, hypertension, infective endocarditis, myocardial infarction (MI)	16	14	1	1
**Tuberculosis (TB):** Extrapulmonary TB, pulmonary TB, TB, TB meningitis	16	2	12	2
**Personal or social problems:** Defaulted, louse infestation, pressure ulcers, social neglect	11	0	9	2
**Endocrine:** Diabetes type 1, diabetes type 2, diabetic ketoacidosis (DKA), metabolic syndrome, thyroid storm	10	9	1	0
**Nephrology:** Chronic kidney disease (CKD), nephritic syndrome	10	8	2	0
**Substance use:** Intravenous drug user (IVDU), substance use disorder (SUD)	10	1	8	1
**Oncological:** Malignancy, malignant melanoma, prostate cancer, space occupying lesion, squamous cell carcinoma	7	2	2	3
**Pulmonary (not related to infections):** Chronic obstructive pulmonary disease (COPD), post-TB bronchiectasis, recurrent pneumothorax	6	4	2	0
**Gastrointestinal tract (GIT):** Abdominal mass, abdominal pain, acute gastroenteritis (AGE), upper gastrointestinal bleeding (UGIB)	5	2	3	0
**Auto-immune:** Systemic lupus erythematosus (SLE), neuro-SLE	4	4	0	0
**Psychological/Psychiatric:** Dementia, psychological disorder, schizophrenia	4	2	2	0
**Thrombotic disease:** Deep vein thrombosis (DVT), pulmonary embolism (PE)	4	3	1	0
**Injury:** Assault, burns, fracture (left hand)	3	0	2	1
**Haematological:** Anaemia, thrombocytopenic purpura (TTP)	2	2	0	0
**Vascular:** gangrene, vasculitis	2	2	0	0
**Urological (not infectious or malignant):** Benign prostatic hyperplasia (BPH)	1	0	1	0
**Immune compromise (not caused by HIV):** Immunocompromised	1	1	0	0

**Total assessments**	**178**	**71**	**77**	**30**

SBAH, Steve Biko Academic Hospital; TRH, Tshwane Rehabilitation Hospital; TDH, Tshwane District Hospital.

#### Personal assessment

During the 80 personal assessments made, 131 items were recorded. Analysed thematically, these categorised individual patients’ psychological well-being (*n* = 77), their relationship to treatment or therapy (*n* = 26) and their levels of function (*n* = 28). As shown in Table 3, negative items relating to patient psychological wellbeing, like anxiety, depression, despair, stress, low motivation, anger and frustration, predominated (78%).

**TABLE 3:** Personal assessment: Number of instances by issue per theme.

Assessment: Personal (131 items)					
Issue	No.	Issue	No.	Issue	No.
Psychological well-being	77	Relationship to diagnosis or treatment therapy	26	Function	28
Denial of condition	11	Poor understanding	3	Unable to talk	3
Depressed/distressed	19	Poor acceptance	4	Unable to take care of self	7
Angry/frustrated	5	Poor adherence	2	Confused	2
Unable to cope with illness	8	Coming to terms	3	Blind	1
Withdrawn, demoralised, demotivated, hopeless	4	Does not want medical attention	1	Immobile	1
Anxious/stressed	6	Has a plan	2	Good mobilised, assisted movement	7
Uncooperative	2	Feels better	6	Mostly or independent	5
Concerned about health	5	Wants help	5	Needs full-time care	2
Feels better	5	-	-	-	-
Wants to go home	5	-	-	-	-
Hopeful, happy	2	-	-	-	-
Motivated	5	-	-	-	-

Those relating to function displayed both negative and positive items, while items recorded about patients’ relationship to treatment and therapy were more positive than negative.

#### Context assessment

Assessments of individual contexts were made with 86 patients (see Table 4). During these, the team recorded 205 items that were categorised thematically into living arrangements (*n* = 62), family circumstances (*n* = 49), environmental factors (*n* = 56) and services (*n* = 38).

**TABLE 4:** Context assessment: Number of instances by issue per theme.

Issues	No.	Issues	No.
**Living arrangements**	**62**	**Family circumstances**	**49**
Lives alone	8	Supportive	15
Homeless	11	Destitute or very poor	2
With family	23	Does not, will not or cannot support	11
With aged parent or grandparent or partner	5	Separated or divorced parents, from partner, from children	7
With siblings	4	Live elsewhere or far	5
Far from family	5	Abusive	2
In old age home	2	Not involved	2
Sent to relative for care	4	Not traceable	3
		Recent death of spouse	2
**Environmental**	**56**	**Services**	**38**
Destitute or very poor	3	Unhappy with length of stay or treatment delay	6
Grant holder or has financial means	4	Multiple admissions or multiple facilities	5
Unemployed or lost job or was working or had piece work	10	Wary of outside help	1
Has dependents or primary breadwinner	8	Not fed for a week – Peg tubes stockout	1
Disability unfriendly facilities, accommodation, location	3	Wants to be discharged	6
Did not finish or can’t go to school	3	Needs or wants placement in care	7
No one to care at home	17	Does not qualify for further care	2
Foreign origin	6	Family supportive in arranging care	4
Xenophobic violence	2	Far from usual place of care	3
		Wants to go back to school or work	3

Table 4 shows that in terms of living arrangements 58% of the items referred to patients living with family, with the remainder reporting patients living alone, being homeless or living far from family. Items under family circumstances show that familial support is the exception (30%) rather than the rule, however.

Most environmental items (68.7%) refer to issues of loss of livelihood, family dependents needing support and the absence of people to provide care. In terms of services, key items relate to multiple admissions and excessive lengths of stay as well as the need to organise care on discharge, in which there are a few instances of family involvement and support.

Although each form of assessment provides insight into the particular issues at hand, in formulating patient downward referral, it is their combination that informs patient-specific plans. As the 14 selected cases in Table 5 illustrate, plans are variously influenced by patients’ clinical conditions, their personal state of well-being and their contextual circumstances.

**TABLE 5:** Three-stage assessment informed down referral planning – 14 selected cases.

Case	Age or sex	Three-stage assessment	Plan and status
1	38 M	**Clinical**: Chronic otitis media, HIV stage 4, treatment failure	**Plan**: Awaiting third-line ARV approval. Application Leratong Hospice
		**Personal**: Defaulted previous treatment. Chronically ill	**Status**: Demised in the ward
		**Context**: Previously employed, good family support, thinks granny can help when discharged	
2	64 M	**Clinical:** Chronic obstructive pulmonary disease (COPD), end-stage, oxygen dependent, benign prostatic hyperplasia (BPH)	**Plan:** Arrange placement oxygen
		**Personal**: Depressed, hopeless	**Status:** Can’t get oxygen without a place to stay, can’t get a place to stay without oxygen
		**Context:** Has SASSA grant. Lived with nephew who cannot accommodate him anymore	
3	21 M	**Clinical:** TB, HIV, substance use disorder (SUD). Defaulted	**Plan**: Hospice placement
		**Personal:** Struggles to take responsibility for health, defaults, demanding	**Status**: Went to Leratong Hospice and COSUP
		**Context:** Steals from mother to buy drugs. Mother can take him back, but apprehensive about adherence	
4	25 M	**Clinical**: Pulmonary TB, HIV diagnosed empirically	**Plan:** Contact reliable house and social worker for housing.
		**Personal:** Cannot survive on street because of illness	**Status:** OT spoke to patient. Made contact with someone from his village. Getting his father’s phone number
		**Context:** Homeless	
5	61 M	**Clinical:** Prostate Ca (stage IV), deep vein thrombosis (DVT)	**Plan**: Dr X to consult with Dr Y about palliative care options
		**Personal:** Denial and concern about seriousness of illness	
		**Context:** Pensioner, originally from W, now lives in Pretoria with employed sister to care for him	
6	6 M	**Clinical:** Guillain-Barré syndrome, now walking with frame. Remarkable recovery, was on life support	**Plan:** Dr Z to get Family Physician number in B District to arrange care
		**Personal:** Playful, friendly and receptive. Seems to relish in being able to walk	
		**Context**: Lives with mother and father in R. Mom needs to go back to work	
7	34 F	**Clinical:** 12 weeks postpartum. Has severe mitral valve stenosis and admitted with warfarin toxicity and heart failure	**Plan:** SBAH Internal Medicine and rehabilitation
		**Personal:** Mourning recent loss of her husband and worries about survival with children	**Status**: Follow-up at Cardiology SBAH. Home visit WBOT. MSF to support
		**Context:** From Malawi, lives alone with her twin babies. Has no financial income and family support. No money for transport for cardiology reviews. Gets medication at X Clinic	
8	55 F	**Clinical:** Cerebrovascular accident (CVA) with vision loss, difficult mobility (left hemiplegia), slow recovery	**Plan:** District rehabilitation team (Mr S), OT needs to be involved
		**Personal:** Frustrated about slow recovery	**Status:** Follow-up at DGMAH
		**Context:** Lives with older sisters in H. Children live in M	
9	39 F	**Clinical**: TB, HIV, newly diagnosed	**Plan:** Dr X to consult SBAH internal medicine, WBOT team leader to arrange team leader visit
		**Personal:** Denial about condition	**Status**: WBOT team leader followed up with team leader. Patient collecting medicines at clinic
		**Context:** Lives in S. Primary breadwinner, has adolescent children, gets family support from sister	
10	31 F	**Clinical**: Infective endocarditis. Intravenous (IV) substance use disorder (heroin)	**Plan:** COSUP to visit patient
		**Context:** Not present, leaves the ward to use substances	**Status:** COSUP saw patient. She declined to be on the programme
		Unable to make assessment	
11	92 F	**Clinical:** acute gastroenteritis (AGE), social neglect, mobile enough to do most activities of daily living (ADLs)	**Plan:** Home visit, Dr H to contact Dr at Daspoort Clinic
		**Personal:** Angry at frailty, feels she cannot handle minor setbacks	**Status**: Daspoort CHW visited house, found it suitable for further care. She was transported to her home
		**Context:** Social neglect: lives alone, son lives close, but often works far away. Does not visit much	
12	27 M	**Clinical**: Diabetes mellitus (DM) type 1, SUD, arm abscess	**Plan:** Follow-up in Inner City: Dr H, COSUP, Rivoningo.
		**Personal:** Motivated to stop using drugs and get DM under control	**Status:** Patient started on insulin, not good control. Premature discharge to Rivoningo. In July readmitted. Discharged in good HGT (blood glucose) control. As of 30 August, good control of DM and SUD
		**Context:** Homeless	
13	56 M	**Clinical**: Skin excoriations, mental healthcare user (MHCU), unknown diagnosis	**Plan:** Placement
		**Personal:** Very withdrawn, non-communicative	**Status**: Discharged to Rivoningo. Still no ID book or traceable family, despite TLF social worker’s best efforts
		**Context:** Homeless. No family traceable. Poor continuity of care: Patient known to WKH but record not available	
14	47 M	**Clinical:** CVA secondary to hypertension and has left hemiplegia	**Plan:** Refer to district rehabilitation to do home training
		**Personal:** Recovering well, walks with a walking stick. He is concerned about how he will get around once he is discharged	
		**Context:** He is married and from Zimbabwe. He lives in a settlement with outdoor mobile toilets. He is a trader. He has to cross the highway to get to his place of business	

ARV, anti-retroviral; CHW, community health worker; CVA, cerebrovascular accident; DGMAH, Dr George Mukhari Academic Hospital; HGT, fasting blood glucose; HIV, human immunodeficiency virus; OT, occupational therapy; SASSA, South African Social Security Agency; TB, tuberculosis; WBOT, ward-based outreach team; WKH, Weskoppies Hospital; SBAH, Steve Biko Academic Hospital; COSUP, Community Oriented Substance Use Programme; MSF, Médecins Sans Frontières (Doctors Without Borders); TLF, Tshwane Leadership Foundation.

### Parallel care-coordination

Parallel care-coordination involved a cross-section of professionals and practitioners from a variety of disciplines and organisations. The UP coordination of care team involved clinicians and health science practitioners, lecturers and researchers from the Departments of Family Medicine, Public Health Medicine, Physiotherapy (PT) and Occupational Therapy (OT). Clinicians and professionals involved in care-coordination at the respective facilities included the nursing manager, ward nursing staff, ward doctors, clinical associates (ClinAs), dieticians, occupational therapists, physiotherapists, speech therapists and social workers. Community-based service providers included the district rehabilitation team manager, WBOT team leaders (OTLs) – who were essential links to home-based follow-up care. Professionals and practitioners from COSUP, palliative (Hospice, Leratong) and homeless care (Tshwane Leadership Foundation – TLF), as well as the migrant health programme (Doctors Without Borders – MSF) were additional partners in linking patients to care beyond the hospital setting.

In addition, in order to put plans into practice the team involved practitioners from other clinical departments and internal services in each of the hospitals, as well as other hospitals, clinics and external social and healthcare service providers. While many were locally situated, this network extended to other provinces.

Plans were made for all patients. Of the 52 patient records where the status of plans were available, 14 patients (27%) were in the wards awaiting further treatment, transfer to another department in the facility or transfer to another hospital. Four patients (7.7%) had been referred to another hospital. Three patients absconded and four patients demised. Twenty seven patients (52%) had been discharged, with seven in 10 being linked to care. While the destination of all discharged patients was not specified, over half were recorded as having been discharged home to family and five went to a hospice, old age home or rehabilitation centre. Two died following discharge.

## Discussion

One of the major stressors in health care is the fact that clinicians and managers are overwhelmed by service demand. In part, this situation arises from a lack of care coordination. As this study shows, it is possible to provide patient care and appropriate solutions that relieve system pressure by conducting clinically led, interprofessional care coordination ward rounds.

As a clinical task in care coordination, the three-stage assessment generates relevant multi-dimensional information that the care team can use to plan parallel coordination.^35^ This study shows that clinical assessments yield essential details about the status of patients’ varied and multimorbid conditions, while personal and contextual assessments put patient care needs and possibilities into perspective. In combination, the three-stage assessment creates an understanding that is greater than the sum of each component. This, in turn, enables the team to plan practical and appropriate onward care.

Three-stage assessments have been advocated as an essential technique in patient-centred management and are an essential family medicine skill.^36^ However, for a number of reasons, they often are not conducted. Apart from the fact that they involve competencies that take time and require practice and perseverance, once done, there are no apparent organisational mechanisms in place to act on them. This study shows how to insert the three-stage assessment into a coordination of care ward round, and transform a century old, ubiquitous practice used in in-patient hospital management^37^ into a vehicle for implementing effective referral.

Care-coordination activities, unlike routine mortality and morbidity meetings, require commitment to practice and review of care provided. The practicality of the process triggers the need to identify and involve people and organisations beyond those directly involved in the clinical care of patients. The study shows that one of the important roles of the care-coordination team is to create networks that enable them to connect patients and their families to a wide range of professionals and service providers within and between facilities and communities. Practically, these networks vary in strength, depth and duration.^13,14^ They are influenced by the viability of the interaction itself, where people are positioned in the network, and whether they see the need to make the choice to cooperate. This said, through active networking and communication, the care coordination team is able to play a critical role in making referral a link-to-care practice in keeping with principles of parallel coordination.^15^

Clinical discharge of patients from one area of care to another is invariably practised as an administrative process. It is filled with formalities, including less than effective patient-held referral letters, as well as considerable uncertainty, with little or no information on patients’ home contexts. This study shows that by making plans based on the three-stage assessment, discharge becomes a richly informed coordinated care decision. Through it patients and their families, as well as facilities and service providers, are informed and can prepare. And just as importantly, it allays clinician disquiet about releasing patients when they are not aware of or confident about where their patients will go and the care they will receive.

## Conclusion

Patient care-coordination involves a set of practices and processes that are intentionally organised and implemented. This study of down-referral describes how this can be implemented by clinically-led, interdisciplinary teams, who conduct care-coordination ward rounds using the three-stage assessment as a tool to generate action plans. Working from a patient-centred community oriented understanding of the people and places required to support the on-going care of each patient, the team facilitates coordination of care by mobilising their family or support system as well as relevant professionals and service providers across sectors and within and between facilities, departments and service levels.

### Recommendations

Four recommendations are derived from this study:

1. Coordination-of-care ward rounds should be instituted to provide clinically informed, patient and family centred continuity of care.
2. Coordination-of-care ward rounds should be routinely conducted by clinician-led interprofessional teams.
3. Care-coordination should be supported by designated, professional linkage-to-care practitioners.
4. Facility- and practice managers should proactively foster networks of practice between health and care professionals, healthcare workers and patients and their families to support parallel coordinating efforts by all members involved.

### Strengths and limitations

The study is based on real time, real patient care management needs. The ward rounds were solution focused, generating practical plans that drew on the best available information and resources.

The composition of the team, both in terms of the actual people who participated and the organisations represented, varied across ward rounds. This may have influenced plans, because during each ward round, tools and assessment recommendations were developed and amended through an iterative process to support a patient-centred, holistic care approach. While not methodologically inconsistent with PAR, this variation could have influenced the results and, as such, is a study limitation.

The population studied comprised patients identified as being stuck in the system and in need of care coordination in the respective wards in level 2, 3 and 4 public facilities. This, together with the purposive patient selection process, means that patient characteristics and assessments are particular to the cases seen and not generalisable to admitted patients in the respective facilities.
